# Genomic Variability of the HCT116 Cell Line Identified Using Oxford Nanopore Sequencing

**DOI:** 10.3390/ijms27135791

**Published:** 2026-06-26

**Authors:** Regina Mikheeva, Pavel Leonov, Maksim Koryukov, Ekaterina Ruleva, Ekaterina Karabut, Andrey Kechin

**Affiliations:** 1 Institute of Chemical Biology and Fundamental Medicine, Novosibirsk 630090, Russiap.leonov@g.nsu.ru (P.L.);; 2 Faculty of Natural Sciences, Novosibirsk State University, Novosibirsk 630090, Russia

**Keywords:** genomic rearrangements, structural variants, single-nucleotide variants, cell line, HCT116, colorectal cancer

## Abstract

HCT116 is a colorectal cancer cell line frequently used in anti-tumor drug development experiments as well as in studies of the molecular machinery of eukaryotic cells. It is well characterized by the presence of several single-nucleotide and short mutations in multiple oncogenes and tumor suppressor genes, including *KRAS*, *PIK3CA*, *MLH1*, *CTNNB1*, *CDKN2A*, *TGFBR2*, and *BRCA2*. However, its landscape of large genomic rearrangements (LGRs) and copy number variants (CNVs) is still far from being fully understood. Therefore, the aim of this study was to identify LGRs and CNVs in several HCT116 cell line samples using Oxford Nanopore sequencing technology, including three samples from the SRA NCBI database, and to compare common and unique variants across all samples. Using the recently developed eLaRodON tool, we identified 22,666 common LGRs, among which more than 70% of tandem duplications and deletions larger than 80 kb were confirmed by CNV analysis. Among LGRs affecting protein-coding sequences, two in-frame rearrangements were identified: a deletion of exons 4–6 and a duplication of exon 10 in the *CCSER1* gene, which encodes a cell division regulator protein. Given its high rearrangement rate in various tumors and the clinical significance of its overexpression, this finding may be potentially useful in future research on this cell line. Regarding differences between samples, we found that LGRs in the laboratory sample and in one of the three SRA NCBI samples occurred more frequently via ALR/Alpha repeats than via Alu repeats, in contrast to common LGRs and those unique to the other samples, a finding that may indicate the presence of unique mechanisms of genomic instability. Thus, this study reveals a broad spectrum of large genomic rearrangements and copy number variants that can be identified in the HCT116 cell line using Oxford Nanopore sequencing, including rearrangements specific to distinct cell line samples.

## 1. Introduction

Today, more than 5000 different tumor cell lines have been obtained from various types of cancer [1]. Their use in in vitro experiments with genome editing technologies or in testing new potential anti-tumor drugs allows researchers to study molecular mechanisms in normal and malignant cells, including metabolic changes and alterations in signaling pathways [2,3]. Newly developed and tested drugs can have substantially different effects on distinct cell lines, and these differences are likely associated with changes in the genetic and epigenetic context of each cell line used [3,4]. This context provides variability in gene expression and protein structure, which manifests as differences in the most important molecular processes in cells, such as cell division, proliferation, and metabolism. Therefore, new anti-tumor drugs under development must be tested on 60 cell lines, as recommended by the National Cancer Institute [5]. Consequently, a deep understanding of all single-nucleotide variants and large genomic rearrangements in the cell line used is an essential requirement for effective new drug design and for studying molecular mechanisms.

While single-nucleotide variants have been extensively studied using short-read sequencing technologies, and our understanding of their representation in cells has already reached the necessary level, the detection of large genomic rearrangements is still under active development [6]. Their identification remains challenging due to the presence of numerous extended repeat sequences in the human genome. Nevertheless, the presence of such rearrangements can substantially alter the repertoire of active genes in cells. Moreover, both tumor cells and cultured cell lines can exhibit significant heterogeneity, including with respect to large genomic rearrangements [7,8].

The HCT116 cell line was obtained from a male patient with colorectal cancer in 1981 [9] and has since been distributed to various research groups worldwide, undergoing many passages. Consequently, different samples of this cell line can substantially differ between research groups, and this variability can influence the results of in vitro experiments using these cells. While the NA12878 (HG001) cell line has gold-standard lists of structural variants and single-nucleotide variants [10], for the HCT116 cell line, only COSMIC copy number alterations and structural variants are known, which obviously do not cover the full spectrum of these genetic changes. At the same time, Oxford Nanopore sequencing technology, like other similar techniques (CycloneSEQ and Qitan Tech), can produce significantly longer sequencing reads and thus provides an opportunity to uncover large genomic rearrangements mediated by extended repeat sequences [11]. Recently, the authors developed a new bioinformatics tool, eLaRodON (https://github.com/aakechin/eLaRodON, accessed on 24 April 2026), which can identify more large genomic rearrangements than other existing tools and allows detection of rearrangements supported by only one sequencing read. Unlike conventional SV callers that merge similar rearrangements through graph-based clustering or self-balancing tree approaches, eLaRodON records each rearrangement as a distinct event and treats multiple split-read fragments as a single sequence, enabling accurate identification of LGRs with two defined boundaries. This approach substantially improves detection of tandem duplications, non-reciprocal translocations, and inversions—variant types that most existing tools can only report as single-boundary breakend (BND) calls. Additionally, eLaRodON systematically evaluates genomic features at each breakpoint, including microhomology and homeology sequences, proximity to repetitive elements, and the presence of novel inserted sequences, providing information relevant for understanding the DNA repair mechanisms involved in rearrangement formation.

Therefore, the aim of this study was to compare the genome structures of several HCT116 cell line samples and to identify genetic variants that are common to all samples studied, as well as those that could potentially explain some metabolic differences between these cells or their responses to chemical drugs.

## 2. Results

### 2.1. Sequencing Yield and NGS Reads Statistics

**Figure 1:**
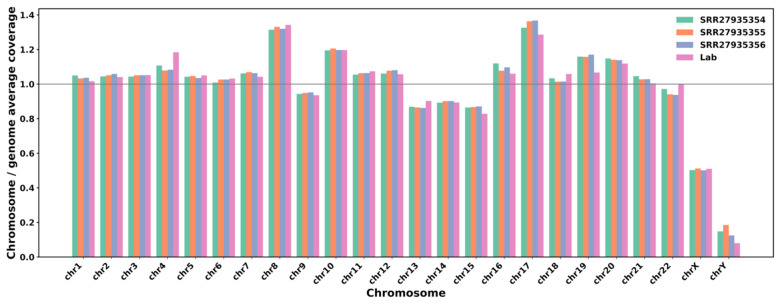
Ratio of mean chromosome coverage to mean genome coverage for all chromosomes of the samples sequenced with Oxford Nanopore technology.

Whole-genome ONT sequencing of the merged dataset from two ONT runs for the laboratory’s HCT116 cell line sample generated a total of 8,759,076 reads, yielding 24.16 Gb of sequence data with a read length N50 of 14,051 bp (mean read length: 2758 bp; standard deviation: 7451 bp) (Table 1). The mean basecall quality score was Q15.3 (median Q18.7), with 93.6% of reads exceeding Q10. The mean genome-wide coverage was 7.16×, which, while below the threshold recommended for SNV calling, is sufficient for split-read-based SV detection at the read lengths obtained. Per-autosome coverage ranged from 5.93× (chr15) to 9.61× (chr8) for the laboratory’s sample, and from 6.59× to 14.96× for the other samples, with similar per-chromosome values across samples (Figure 1). Coverage values for chromosomes X and Y were 3.65× and 0.57×, respectively, which is consistent with the male origin of the HCT116 cell line.

### 2.2. Single Nucleotide Variants and Short Insertions and Deletions

To identify SNVs and short insertions and deletions (InDels) common to all samples and sample-specific ones, we applied Clair3 which yielded a total of 5,459,060–7,022,823 raw point mutations and short InDels per sample with minimal value for Illumina sequencing data. Considering that the Illumina sequencing technology has the lowest error rate, we filtered out the Illumina’s variants with the following thresholds: number of reads with alternative allele ≥ 6, variant allele frequency (VAF) ≥ 0.2. The Illumina data were used solely to establish an independent reference set of high-confidence point mutations for cross-platform comparison; ONT variant filtering was performed independently based on the thresholds described below. Such filtering left 4,480,712 variants for the subsequent analysis. Among them, 3,735,607 variants were identified simultaneously in all HCT116 samples studied with 11,559 variants probably affecting proteins’ amino acid sequences with 1557 written in the COSMIC database for the HCT116 cell line (Supplementary Table S1).

Among the common variants all the known point pathogenic mutations for the HCT116 cell line were successfully identified in almost all samples. The canonical oncogenic driver mutations characteristic of HCT116—*KRAS* p.G13D (c.38G>A), *PIK3CA* p.H1047R (c.3140A>G), *CTNNB1* p.S45del (c.133_135delTCT), biallelic inactivation of *MLH1* (c.755C>A, p.S252*), frameshift alterations in *TGFBR2* (c.383delA, p.Lys153fs), *CDKN2A* (c.97delG p.Glu33fs and c.68dupG p.Arg24fs), *BRCA2* (c.8021dupA, p.I2672fs), and *PPM1D* c.1349delT. *BRCA2* c.8021dupA and *PPM1D* c.1349delT were not identified in SRR27935356 and SRR27935355 samples, respectively. For the SRR27935356 sample, it was due to the low total coverage value (three reads) in that region. However, for SRR27935355, the deletion was missed due to the low VAF (only two of 16 reads contained the alternative allele), which may indicate coverage bias or a clonal shift in the genetic background of this sample. At the same time, mutations in the *TGFBR2* and *CDKN2A* have not been written in the COSMIC database (version 103) for the HCT116 cell line.

Cross-platform concordance was evaluated by comparing each ONT sample against the Illumina dataset using bcftools isec. For SNVs, the mean precision (proportion of ONT calls confirmed by Illumina) was 77.9% and the mean recall (proportion of Illumina calls detected in ONT) was 92.8%. InDel concordance was lower (mean precision 46.8%, recall 74.9%), consistent with the known susceptibility of nanopore sequencing to systematic errors in homopolymer regions. The laboratory sample data showed the lowest concordance values (SNV recall 83.9%), consistent with its lower sequencing depth (7.16×).

### 2.3. Large Genomic Rearrangements

To investigate large genomic rearrangements in the HCT116 cell line samples, we began by examining their karyotypes and chromosomal abnormalities exceeding 10 Mb in size. The karyotype of the HCT116 cell line has been previously described with varying levels of detail. In the review by Turid Knutsen and colleagues, it was reported as 45,X,Y,der(4)t(4;17)(q3?;?q21), del(9)(q11), der(10)dup(10)(q23.1q26.1)t(10;16)(q26.1;q23), der(16)t(8;16)(q13;pter), der(18)(:4q3?::17q?22→17q21.3::18pter→18qter) [12]. In the study by Alisa Morshneva and coauthors, the HCT116 cell line was reported to have a 46,XY,der(10)dup(10)(q22q23),der(8),t(8;16)(q13;p13.3),der(18)t(17;18)(q11.2;p11.2) karyotype [13]. Oxford Nanopore sequencing technology theoretically provides an opportunity to identify the exact boundaries of genomic rearrangements. Therefore, we attempted to identify the listed rearrangements among the LGRs and CNVs detected. Among four interchromosomal translocations, only one was identified with exact boundaries as BND_TRL, supported by 3–6 reads (Table 2). For large chromosomal duplications and deletions, CNV boundaries were determined only using the ONT-spectre tool without confirmation with eLaRodON.

In addition to large chromosomal rearrangements, smaller deletions and duplications ranging in size from tens of thousands to several million base pairs were identified using ONT-spectre, some of which were confirmed by LGR boundaries from eLaRodON (Table 3). Although ONT-spectre yielded more CNVs than LGRs of similar size, including both common and sample-specific CNVs (Supplementary Table S2), confirmation of these events will require higher depth of human genome coverage or the application of alternative methods such as droplet digital PCR. For chromosomes with highest mean coverage (chr17, chr8, and chr10), the number of duplicated genomic regions was also the highest (Figure 1 and Supplementary Table S2).

The next group of LGRs comprised variants identified with eLaRodON simultaneously in all samples as well as sample-specific variants (Supplementary Tables S3–S7). The total number of common LGRs of size 50 bp or larger was 22,666 variants (8723 deletions, 248 tandem duplications, 52 inversions, 138 interchromosomal translocations, and 13,505 insertions). Some of these were confirmed by CNV analysis using ONT-spectre or by depth-of-coverage analysis using mosdepth. Three BND_DEL rearrangements larger than 2–27 Mb were not confirmed by coverage analysis, suggesting possible translocation of the regions instead of deletion. Although the VAF values for common LGRs were correlated between samples—in particular for LGRs with a depth of coverage of at least ten (Supplementary Figure S2)—high variability in the number of supporting reads and in VAF values was observed across samples.

**Figure 2:**
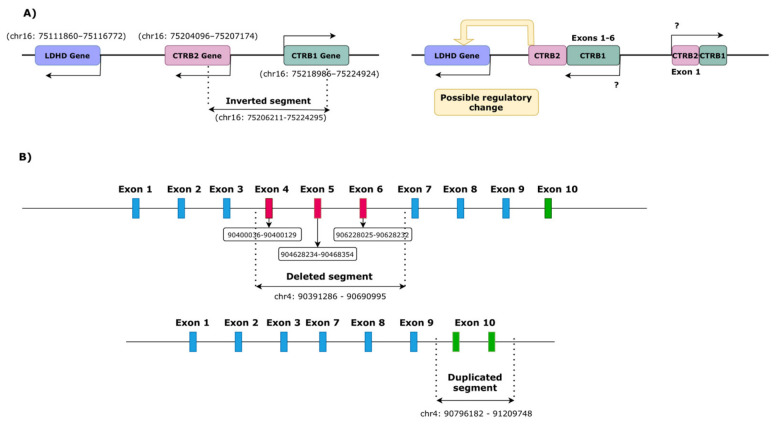
Examples of LGRs affecting possible regulatory region of lactate dehydrogenase D (*LDHD*) gene (**A**) and *CCSER1* gene encoding cell division regulatory protein (**B**). Arrows indicate the transcription start sites, and the question mark denotes the unknown possibility of expression of the chimeric genes formed.

Among the LGRs common to all samples studied, 8791 (38.8%) and 353 (1.6%) overlapped with genes and exons, respectively. The percentage of such LGRs among sample-specific variants was similar: 34–35% for SRR27935354 and the laboratory’s sample and 44–50% for SRR27935355 and SRR27935356. Examples of three LGRs that possibly affect the structure or regulation of genes are shown in Figure 2. One large inversion of 18,128 bp changed the putative regulatory region of the lactate dehydrogenase D (LDHD) gene (about 90 Kb downstream of the gene start, Figure 2A). Another included a heterozygous deletion of exons 4–6 of the *CCSER1* gene and a simultaneous tandem duplication of exon 10 with adjacent intronic regions (Figure 2B). Both variants did not affect the protein coding frame; therefore, a functional protein with altered properties is likely to be expressed. Considering that this gene encodes the FAM190A protein, which regulates cell division [14,15], these variants could substantially affect cellular mechanisms of mitosis.

Although all the studied HCT116 cell line samples shared many common LGRs, sample-specific variants were also identified. For example, 482 LGRs (280 insertions, 122 tandem duplications, 73 deletions, 4 inversions, and 3 interchromosomal translocations) were identified in the laboratory’s sample (Supplementary Table S7). Among these, 167 LGRs overlapped with genes, and 14 were likely to alter protein-coding sequences.

**Figure 3:**
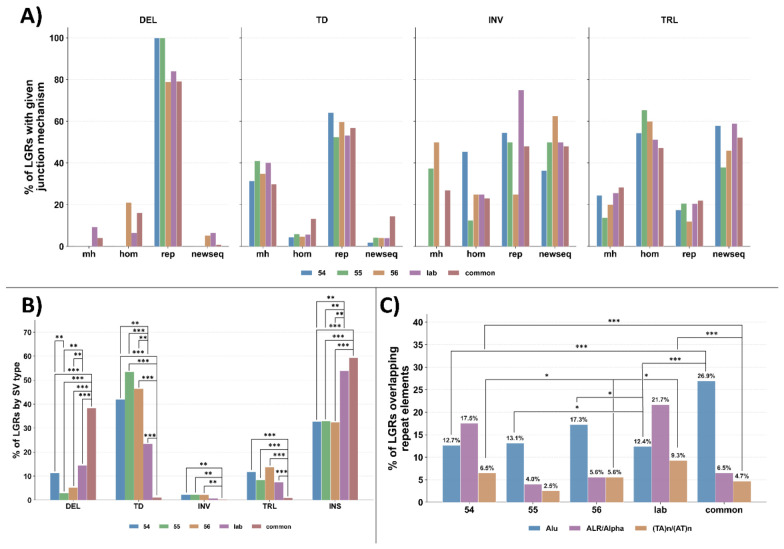
Comparison of LGRs by their ratio and type of junction boundaries. (**A**) Type of junction boundary homology: microhomology (mh), homeology (hom), or repeat (rep). “Newseq” indicates the presence of a sequence of unknown origin between two joined sequences. This category does not overlap with other junction types; i.e., a novel sequence can be present together with microhomology or homeology, or in LGRs without any homology. (**B**) Distribution of different types of LGRs among all samples studied. DEL—deletions, TD—tandem duplications, INV—inversions, TRL—interchromosomal translocations, INS—insertions. (**C**) Distribution of LGRs by the type of repeat sequence through which the LGR occurred. Only Alu repeats, ALR/Alpha, and (TA)n with (AT)n are shown. Asterisks indicate statistical significance: * *p* < 0.05, ** *p* < 0.01, *** *p* < 0.001 (Fisher’s exact test with Bonferroni correction for multiple comparisons).

To assess possible differences in the mechanisms of mutagenesis, we compared the structures of common and sample-specific LGRs by their type ratios and junction boundary features, particularly the presence of microhomology or homeology and the distance from repeat sequences to the breakpoint (Figure 3). Based on the distribution of LGR types, the most striking difference was observed between common LGRs and sample-specific ones (Figure 3B). Whereas LGRs present in all samples were predominantly insertions (59%) and deletions (38%), tandem duplications and interchromosomal translocations constituted a much larger share among sample-specific LGRs: 23–53% and 8–14%, respectively (p-values ranged from 2 × 10^−238^ to 5 × 10^−110^, Fisher’s exact test). The proportion of tandem duplications among all LGR types was also significantly lower in the laboratory’s sample compared with the other samples (*p*-values ranged from 2 × 10^−19^ to 5 × 10^−10^, Fisher’s exact test). Although differences in the presence of homologous sequences between junction boundaries were also observed between LGR groups, they were not statistically significant.

By LGR type ratios, the sample most similar to the laboratory’s sample was SRR27935354. This similarity was also maintained when comparing repeat sequences located at LGR boundaries (Figure 3C). The difference between LGRs common to all samples and sample-specific LGRs was statistically significant only for SRR27935354 and the laboratory’s sample with respect to the proportion of LGRs occurring via Alu repeats (*p* = 6 × 10^−15^ and 2 × 10^−17^, respectively). Thus, the similarity between SRR27935354 and the laboratory’s sample was confirmed in two independent comparisons of LGR structure.

## 3. Discussion

In this study, we performed an in-depth comparison of several HCT116 cell line samples sequenced using Oxford Nanopore technology. By combining the results of SV and CNV detection across all samples and applying eLaRodON—a tool recently developed by the authors—we were able to identify numerous LGRs common to all studied samples, as well as several sample-specific variants. Most of these LGRs and CNVs are described for this cell line for the first time, which was made possible by the unique algorithm of eLaRodON that reports all LGRs supported by even a single sequencing read. The identification of LGRs is significantly complicated by the low probability that a sequencing read will span an LGR in a way that reaches the end of the repeat element [16,17]. If we had not included LGRs supported by only one read, we would have missed 7710 (34%) of the LGRs common to all samples (Supplementary Table S3). Consequently, the number of identified LGRs would have been close to that typically reported for human DNA samples [18,19,20,21,22]. Moreover, many potential common LGRs were excluded from both the common and sample-specific lists because they were detected in only two or three samples. Therefore, the true number of common LGRs may be even higher than we report here.

Among the 22,666 common LGRs, only 42.3% were supported by three or more reads in all four samples simultaneously. The remaining LGRs had fewer than three supporting reads in at least one sample, with the proportion of single-read-supported LGRs being highest in the laboratory sample (16.7%), consistent with its lower sequencing depth (7.16×). False-positive LGRs were controlled by requiring independent detection in all four samples, applying a VAF threshold of ≥0.3, filtering out LGRs within 100 bp of genome assembly gaps, and using eLaRodON’s quality scoring system that evaluates breakpoint characteristics and proximity to repetitive elements.

Taking into account LGRs with only one supporting read, it would be of great interest to study subclonal LGRs, whose detection is limited not only by the complications mentioned above but also by their rare occurrence among all sequencing reads obtained [23]. Consequently, if a supporting read threshold greater than 1–2 is applied, coverage of approximately 1000× is required to detect an LGR present in only 5% of cells. Achieving such coverage would require about 200 Oxford Nanopore flow cells per sample. Nevertheless, alternative approaches are currently required to validate LGRs with single supporting reads, and the identification of subclonal LGRs will be the focus of our future research.

Although it may seem counterintuitive that we applied a higher alternative allele depth threshold for SNVs and short InDels (five or six reads) than for LGRs (three reads), this choice is explained by the higher probability of obtaining a random false-positive SNV compared to a false-positive LGR. During sequencing, the probability of an incorrectly called base occurring at the same position in two or three reads across the whole genome is much higher than that of an incorrect junction of two DNA fragments at the same chromosomal position. Therefore, we believe that our choice of thresholds is appropriate, and similar thresholds have been used in other studies [24,25].

The orthogonal confirmation of identified LGRs is a complicated task, accompanied by difficult primer design to avoid repeat sequences, and clearly cannot be carried out using only short-read sequencing techniques, as has been done in some other studies [26,27]. While large deletions and duplications can be confirmed by evaluating coverage values for the corresponding genomic regions—as was done in this study—other types of LGRs, such as inversions or translocations, require the use of other techniques. Moreover, although more than 70% of LGRs of size 80 kb or larger were confirmed by changes in depth of coverage, there were large deletions and duplications not confirmed by coverage value changes, as well as many CNVs not confirmed by LGRs. The former inconsistencies may be associated with the subclonal nature of those LGRs [28], whereas the latter may be caused by complications in LGR detection discussed above. Furthermore, at the sequencing depths used in this study (7–11×), read depth-based CNV detection is susceptible to false positives arising from stochastic fluctuations in coverage, which may account for a proportion of the CNVs that lack LGR confirmation.

The concordance between the two approaches is asymmetric: more than 70% of LGRs ≥ 80 kb were confirmed by CNV analysis, whereas a substantially lower proportion of CNVs were confirmed by LGRs. This asymmetry is expected and reflects fundamental differences in their detection principles. CNV calling from read depth analysis can identify copy number changes even when no individual read spans the breakpoint, whereas LGR detection requires junction-spanning reads that must traverse and exit flanking repeat elements. This limitation is particularly relevant for breakpoints located within long repetitive sequences, where the probability of obtaining a spanning read decreases with repeat length and sequencing depth.

To provide orthogonal validation of CNV calls, read-depth-based CNV calling was performed on the Illumina dataset using Control-FREEC, confirming 72.7% of ONT-spectre CNVs (62.5% of deletions and 95.2% of duplications). In the reverse direction, 15.6% of Control-FREEC CNVs were confirmed by ONT-spectre, which is expected given the higher Illumina coverage (17.7×) relative to ONT (7–11×).

Regarding the total of 22,666 common LGRs, this number is partly explained by eLaRodON’s design, which records each rearrangement event individually rather than merging overlapping calls through de novo assembly, as some other tools do. This approach is important for studying rearrangement formation mechanisms but yields higher variant counts. Additionally, insertions constitute 59.6% of all common LGRs (13,505 of 22,666), a variant class that is frequently underreported by other SV callers. Finally, the requirement that each LGR be independently detected in all four samples provides strong protection against false positives, as the probability of an artifactual chimeric read occurring at the same coordinates across independent experiments is negligibly low.

The structural differences observed between HCT116 samples are likely attributable to a combination of biological and technical factors. On the biological side, independent propagation of the same cell line in different laboratories is known to result in substantial genomic divergence through clonal selection and genetic drift [3,29,30]. This process is expected to be particularly pronounced in the HCT116 cell line, which harbors a mismatch repair deficiency due to MLH1 promoter methylation, predisposing it to elevated mutation rates. However, comparative analyses of multiple cell lines have shown that HCT116 exhibits relatively high genomic stability compared to other widely used cancer cell lines such as HeLa or MCF7, with SNV concordance exceeding 98% across independent RNA-seq datasets [31]. This relative stability, combined with the well-characterized heterogeneity of HCT116 subclones [7], suggests that while passage-related drift is expected, HCT116 may be less susceptible to rapid genomic diversification than other commonly used cell lines. The passage history of the samples compared in this study is largely unknown: the laboratory sample was obtained after at least 10 passages at the providing institution, and the exact passage numbers for the publicly available datasets were not reported. Given the well-documented impact of passage number on genomic profiles of cultured cell lines [3,29,30], the absence of this information represents a limitation of the biological interpretation of inter-sample differences. On the technical side, differences in sequencing depth (ranging from 7.16× to 11.28×) directly affect the sensitivity of LGR detection, particularly for low-frequency variants.

An additional source of technical variability arises from the unknown basecalling models and settings used for the publicly available ONT datasets. Different basecalling algorithms and model versions can produce substantially different read accuracies, which may affect variant calling, particularly for SNVs and short InDels. The impact on LGR detection is expected to be more limited, as eLaRodON identifies rearrangements based on the alignment structure of split reads rather than on individual base-call accuracy. Nevertheless, this heterogeneity in data preprocessing represents a limitation of cross-sample comparisons performed in this study. Distinguishing biological variability from technical artifacts remains challenging at the sequencing depths used, and future analyses with higher coverage and documented passage histories may help resolve this question.

Among the LGRs common to all HCT116 cell line samples studied, we describe in more detail two LGRs that, in our opinion, merit attention. The first is an inversion near the lactate dehydrogenase D gene. The encoded enzyme catalyzes the conversion of D-lactate to pyruvate, as well as other D-2-hydroxyacids [32]. Its elevated level in colorectal cancer is associated with poor overall survival [33], and changes in its expression can be caused by various molecular mechanisms, including the potential ones identified here for the HCT116 cell line. The second LGR is a complex rearrangement: an in-frame large deletion of three exons accompanied by an in-frame duplication of another exon in the *CCSER1* gene, which encodes a cell division regulator [14,15]. Deletions of certain exons of this gene have already been described in the literature and may lead to its hyperexpression, thereby enhancing proliferation and genomic instability [15]. Other studies, however, suggest that altered *CCSER1* could be considered a target neoantigen for immunotherapy [34]. Nevertheless, to the best of our knowledge, a dual rearrangement of the type identified in our study has not been reported previously.

A potentially useful difference between the cell line samples was identified regarding the junction boundary structure of sample-specific LGRs. For two samples—the laboratory sample of the HCT116 cell line and SRR27935354—we observed a prevalence of LGRs mediated by ALR/Alpha repeat sequences rather than Alu repeats, in contrast to what was observed for the other samples and for common LGRs. This suggests that additional mechanisms of genomic instability, beyond the known mismatch-repair deficiency, may have been acquired in these two samples during previous passages. Given that ALR/Alpha repeats are predominantly located in centromeric and pericentromeric regions, a higher rate of aneuploidy and chromosomal instability could be expected for these samples [35,36].

## 4. Materials and Methods

### 4.1. Cell Culture and DNA Isolation

The experiments were performed using the HCT116 cell line, obtained from the collection of the Institute of Cytology and Genetics, Siberian Branch of the Russian Academy of Sciences (Novosibirsk, Russia). Mycoplasma-tested HCT116 cells were maintained in Dulbecco’s Modified Eagle’s Medium (PanEco, Moscow, Russia) supplemented with 10% (*v*/*v*) heat-inactivated fetal bovine serum (Thermo Fisher Scientific, Waltham, MA, USA), 2 mM L-glutamine (Capricorn Scientific, Ebsdorfergrund, Germany), 100 U/mL penicillin (PanEco, Moscow, Russia), and 100 μg/mL streptomycin (PanEco, Moscow, Russia) under standard conditions at 37 °C in a humidified atmosphere containing 5% CO_2_.

Genomic DNA was extracted from cells using the SolPure HW DNA extraction kit (Magen Biotechnology Co., Ltd., Guangzhou, China) according to the manufacturer’s protocol for genomic DNA isolation from cultured cell lines. DNA quality was assessed using an ND-100C spectrophotometer (Hangzhou Miu Instrument Co., Ltd., Hangzhou, China), and DNA concentration was quantified using a Qubit fluorometer (Thermo Fisher Scientific, Waltham, MA, USA) with the SynQuant BR DNA-100 reagent kit (Syntol, Moscow, Russia) in accordance with the manufacturer’s protocol.

The laboratory HCT116 sample was obtained from the cell bank after at least 10 passages at the institution. However, the exact number of earlier passages has not been recorded.

### 4.2. DNA Library Preparation and Sequencing

Sequencing libraries were prepared using the NEBNext Ultra II kit (New England Biolabs, Ipswich, MA, USA) and the ligation sequencing DNA kit V14 (SQK-LSK114, Oxford Nanopore Technologies, Oxford, UK) according to the manufacturers’ protocols. The input DNA concentration was 291 ng/µL, yielding a final amount of 1 µg per reaction. Sequencing was performed on the MinION Mk1B platform (Oxford Nanopore Technologies, Oxford, UK) using a R10.4.1 flow cell (FLO-MIN114) following the manufacturer’s protocol. Two sequencing runs were conducted, each lasting 72 h.

### 4.3. External Sequencing Data

For comparative analysis, publicly available whole-genome sequencing datasets were retrieved from the NCBI Sequence Read Archive (SRA). ONT sequencing data for the HCT116 cell line were obtained under accession numbers SRR27935354, SRR27935355, and SRR27935356 (BioProject PRJNA1075154). Pre-basecalled FASTQ files were downloaded using the SRA Toolkit [37]. The basecalling model and settings used by the original data submitters were not specified in the BioProject metadata; no additional basecalling was performed on these datasets. The downloaded reads were processed using the same bioinformatics pipeline described below to ensure methodological consistency across all comparisons. For independent validation of the identified point variants, an Illumina whole-genome sequencing dataset for the HCT116 cell line was obtained from the NCBI SRA under accession number SRR8639145 (BioProject PRJNA523380). This dataset was used exclusively for cross-platform validation of SNVs and short InDels and was not employed for filtering or prioritizing ONT-derived variants.

### 4.4. Sequencing Data Analysis

After sequencing, base calling was performed using Dorado (version 1.3.0; Oxford Nanopore Technologies, Oxford, UK) with default parameters. Reads were subsequently aligned to the human reference genome version GRCh38.p14 using minimap2 (version 2.24-r1122) [38]. Point mutations were called with the Clair3 tool (v2.0.0) [39]. Large genomic rearrangements were identified with eLaRodON (https://github.com/aakechin/eLaRodON, accessed on 24 April 2026) and filtered based on the number of supporting reads (at least three supporting reads in at least one of the samples), variant allele frequency (at least 0.3 in at least one of the samples), and proximity to the hg38 genome assembly gaps. Any LGR located within 100 bp of an assembly gap was filtered out. Copy number variations were called using mosdepth (v0.3.12) [40] and ONT-spectre (v0.3.3) (https://github.com/nanoporetech/ont-spectre, accessed on 24 April 2026) tools. Sequencing data quality was assessed using samtools (version 1.23.1) [41], Nanoplot (version 1.46.2) [42], and FastQC (version 0.12.1).

The Illumina sequencing data were mapped to the human reference genome using BWA-MEM2 [43], and variant calling was performed using Clair3 [39] with the paired-end Illumina model. Clair3 was chosen over alternative short-read variant callers (e.g., GATK HaplotypeCaller, DeepVariant) to maintain methodological consistency with the ONT analysis pipeline and to minimize inter-tool variability in cross-platform comparisons. To determine the impact of the variants on the encoded protein sequence, the snpEff (v5.4c) tool was used [44].

Cross-platform concordance of SNVs and short InDels was evaluated by comparing normalized variant calls (bcftools norm [45]) for each ONT sample against the Illumina dataset. Variants were split into SNVs and InDels, and intersection analysis was performed using bcftools isec. Precision was defined as the proportion of ONT variant calls confirmed by the Illumina dataset, and recall as the proportion of Illumina calls detected in the ONT data.

Copy number variation calling on the Illumina dataset was performed using Control-FREEC v11.6 [46] with a window size of 50 kb, a ploidy of 2, and GC content normalization. CNVs smaller than 10 kb were excluded. The resulting calls were compared with ONT-spectre CNVs using positional tolerance of 400,000 bp, size tolerance of 500,000 bp, or reciprocal overlap of at least 30%.

### 4.5. Comparison of Large Genomic Rearrangements and Copy Number Variations

To identify LGRs common to all the HCT116 samples, the VCF files produced by eLaRodON were compared according to the following rules. Two variants were considered identical across samples if their types and junction directions matched, and if their genomic coordinates and sizes differed by no more than 10% or 200 bp (whichever was more stringent); for inter-chromosomal rearrangements (BND_TRL), the positional tolerance was extended to 500 bp. Such threshold was chosen based on the possible inaccuracy in the alignment of large reads.

To identify LGRs corresponding to copy number variations (CNVs), we attempted to find deletions or tandem duplications with the same or broader (but with a size difference of less than 500,000 bp compared to the CNV size) coordinates and with a positional difference relative to the CNV of less than 400,000 bp. These parameters were determined by comparing CNVs for which LGRs with a confident number of supporting reads (at least three) had been identified. The results are presented in Supplementary Figure S1.

For the analysis of junction boundaries, an LGR was considered to arise through repeat sequences if the two repeat sequences at the boundaries were identical.

### 4.6. Statistical Analysis

For statistical analysis, the SciPy (v1.17.1) [47] and NumPy (v2.4.4) [48] Python (v3.12.3) modules were used. To test the statistical significance of differences in the ratios of the number of LGRs between samples, Fisher’s exact test with Bonferroni correction for multiple comparisons was applied.

## Figures and Tables

**Table 1:** Summary of sequencing metrics for the HCT116 cell line samples analyzed in this study. Lab—the laboratory sample of the HCT116 cell line. 54—SRR27935354, 55—SRR27935355, 56—SRR27935356.

	Lab	54	55	56	SRR8639145
Sequencing Platform	ONT	ONT	ONT	ONT	Illumina
Total Reads, millions	8.76	3.20	2.00	2.85	640.98
N50, bases	14,051	13,302	15,245	13,767	101
Mean Read Length	2758	11,268	11,984	11,139	93
Mapping rate, %	100.00	99.96	99.95	99.98	97.84
Total Yield, Gb	24.16	29.9	20.3	27.2	59.8
Mean Coverage	7.16	11.28	7.62	10.2	17.7
GC Content, %	41.0	41.3	41.4	41.5	43.2

**Table 2:** Large chromosomal rearrangements and CNVs identified with eLaRodON and/or ONT-spectre tools.

Chromosomal Rearrangements	Coordinates	Samples
der(4)t(4;17)(q3?;?q21)	not detected	
der(10)t(10;16)(q26.1;q23)	not detected	
der(16)t(8;16)(q13;pter) der(8),t(8;16)(q13;p13.3)	chr8:66472203	54, 55, 56, lab
	chr16:680431	
der(18)(:4q3?::17q?22→17q21.3::18pter→18qter der(18)t(17;18)(q11.2;p11.2)	not detected	
del(9)(q11)	44,061,000–45,517,000	54, 55, 56, lab
der(10)dup(10)(q23.1q26.1)	duplication of several regions from ~98,529,000 to ~125,912,000	54, 55, 56, lab
der(10)dup(10)(q22q23)	68,800,000–95,300,000	54, 55, 56, lab

**Table 3:** Copy number variations (CNVs) identified in all HCT116 cell line samples, some of which were confirmed as large genomic rearrangements (LGRs) using the eLaRodON tool. CNVs previously reported in the COSMIC database are highlighted in bold. Coordinates of CNVs specified from the COSMIC database are shown in italic.

Chr	Start	End	Length	Type	Zyg.	Samples	Method
chr2	140,940,220	141,441,937	501,718	Del	Hetero	All	LGR, CNV
chr2	204,625,353	204,930,317	304,965	Del	Hetero	All	LGR, CNV
chr3	60,235,172	60,404,776	169,605	Del	Hetero	All	LGR, CNV
chr3	60,604,285	60,843,856	239,572	Del	Hetero	All	LGR, CNV
chr3	91,555,000	91,713,000	158,000	Del	Homo	All	CNV
chr4	90,391,268	90,690,995	299,728	Del	Hetero	All	LGR, CNV
chr6	1,783,057	2,034,965	251,909	Del	Hetero	All	LGR, CNV
chr6	2,030,628	2,268,487	237,860	Del	Hetero	All	LGR, CNV
chr6	20,375,926	20,624,595	248,670	Dup	Hetero	All	LGR, CNV
chr6	58,447,373	58,798,642	351,270	Del	Homo	All	LGR, CNV
chr16	6,165,928	7,090,949	925,022	Del	Hetero	All	LGR, CNV
**chr16**	**6,172,438**	**6,884,622**	**712,185**	**Del**	**Homo**	**All**	**LGR, CNV**
chr16	78,228,812	78,442,742	213,931	Del	Hetero	All	LGR, CNV
**chr17**	***18,455,633***	***18,561,273***	**105,641**	**Del**	**Homo**	**All**	**CNV**
chr17	24,250,000	24,899,000	649,000	Del	Homo	All	CNV
chr17	25,015,000	26,509,000	1,494,000	Del	Homo	All	CNV
chr19	20,490,322	20,841,029	350,708	Del	Hetero	All	LGR, CNV
chr19	53,990,998	55,113,922	1,122,925	Del	Hetero	All	LGR, CNV
chr20	29,865,027	30,945,361	1,080,335	Del	Homo	All	LGR, CNV
chr22	18,204,639	18,893,221	688,583	Del	Homo	All	LGR, CNV
chrX	29,364,601	29,501,641	137,041	Dup	Homo	All	LGR, CNV
**chrX**	**29,523,494**	**29,945,784**	**422,294**	**Del**	**Homo**	**All**	**LGR, CNV**

## Data Availability

The NCBI SRA data used in the study can be accessed through BioProject PRJNA1075154. The lists of large genomic rearrangements and copy number variants identified are available in the Supplementary Tables. The raw sequencing data obtained in this study is available upon reasonable request.

## Supplementary Materials

The following supporting information can be downloaded at https://www.mdpi.com/article/10.3390/ijms27135791/s1.

## Abbreviations

The following abbreviations are used in this article:

CNVs	Copy number variants
InDels	Insertions and deletions
LGRs	Large genomic rearrangements
RSF	Russian Science Foundation
SNVs	Single Nucleotide Variants
SRA	Sequence Read Archive
VAF	Variant allele frequency
